# Effect of Supplementation with Black Soldier Fly Extract on Intestinal Function in Piglets Infected with Porcine Epidemic Diarrhea Virus

**DOI:** 10.3390/ani14101512

**Published:** 2024-05-20

**Authors:** Chenmin Yu, Mengjun Wu, Lanyuan Sun, Hanxiao Li, Zhaoyang Xu, Qian Zhang, Dan Yi, Lei Wang, Di Zhao, Yongqing Hou, Tao Wu

**Affiliations:** Hubei Key Laboratory of Animal Nutrition and Feed Science, Wuhan Polytechnic University, Wuhan 430023, China; yucm0430@163.com (C.Y.); wumengjun93@163.com (M.W.); sunlanyuan2021@163.com (L.S.); 666lihanxiao@gmail.com (H.L.); 18437978537@163.com (Z.X.); zhangqian870305@126.com (Q.Z.); yidan810204@163.com (D.Y.); wanglei_wh@aliyun.com (L.W.); zhaodi24@163.com (D.Z.); houyq@aliyun.com (Y.H.)

**Keywords:** black soldier fly extract, piglets, porcine epidemic diarrhea virus, intestinal function, antioxidant capacity

## Abstract

**Simple Summary:**

Porcine epidemic diarrhea (PED) is caused by a coronavirus that poses a serious threat to the global pig industry. Black soldier fly is a very high-quality protein raw material that could serve as a functional feed additive to improve intestinal health in piglets. The study aimed to evaluate the effects of black soldier fly extract (BFE) on growth performance, plasma biochemical parameters, antioxidant capacity and antiviral genes in PEDV-infected piglets. The results showed that the addition of 500 mg/kg BW BFE to the diet could improve the intestinal function of piglets and activate the immune system after PEDV infection.

**Abstract:**

Porcine epidemic diarrhea virus (PEDV) has developed as a global problem for the pig business, resulting in significant financial losses. Black soldier fly extract (BFE) has been proven to improve intestinal growth in pigs after weaning. Consequently, the goal of the present investigation was to explore the effects of BFE supplementation on intestinal function in PEDV-infected piglets. Eighteen piglets were randomly allocated to three groups: control, PEDV, and BFE + PEDV. The piglets in the BFE + PEDV group received 500 mg/kg BW of BFE orally for seven days from day 4 to 10 of the study. On day 9 of the study, six pigs from each group received either clean saline or PEDV solution at a dosage of 10^6^ TCID_50_ (50% tissue culture infectious dose) per pig. On day 11, samples of blood and intestine were taken for additional investigation. The results indicated a significant decrease in the average daily gain (ADG) of piglets infected with PEDV (*p* < 0.05). Additionally, PEDV infection led to an alteration of blood indexes and a reduction in plasma D-xylose concentration and villi height in the small intestine, while it increased plasma diamine oxidase activity and small intestinal crypt depth in piglets (*p* < 0.05). The PEDV infection significantly reduced antioxidant enzyme activity in plasma and the gut, including total superoxide dismutase and catalase, while increasing contents of oxidation-relevant products such as malondialdehyde and hydrogen peroxide in piglets. Moreover, PEDV infection increased the mRNA expression level of antiviral-related genes (*p* < 0.05). Nutritional supplementation with BFE improved intestinal histomorphological indicators and reduced oxidative stress produced by PEDV infection in piglets. Interestingly, BFE could significantly promote the mRNA expression level of antiviral-related genes in the ileum (*p* < 0.05). Overall, the preliminary results suggest that dietary BFE could improve intestinal function in piglets after PEDV infection. Currently, the findings put a spotlight on the role of BFE in the prevention and treatment of PED in piglets.

## 1. Introduction

Porcine epidemic diarrhea virus (PEDV), an animal virus of the species Alphacoronavirus in the Coronaviridae family of the order Nidovirales, has been regarded as a severe danger to the pig business globally [1,2,3]. PEDV attacks villous epithelial cells throughout the gut, causing immediate severe atrophic enteritis and viremia, culminating in severe diarrhea and vomiting, followed by widespread dehydration, which is the major cause of mortality in suckling piglets [4]. Vaccines are typically utilized for shielding piglets from PEDV infection; however, there are no identified drugs or treatments specifically designed to prevent or cure PEDV infection [5]. Therefore, there is an increasing demand for the development of alternative agents against PEDV that operate through a distinct mechanism of action.

Insects are a very high-quality protein raw ingredient and are considered a promising functional feed additive. Black soldier fly, a saprophytic hydrofidae bug, can absorb both chicken dung and home garbage [6]. Its larvae are high in protein, amino acids, lauric acid, and minerals, making them a high-quality feed item. It has been found that supplementation with 1% black soldier fly pulp can enhance the transcription, digestive enzyme activity, liver antioxidant capacity and immune performance of intestinal tight junction proteins in juvenile golden pomfret fish, and inhibit pathogenic microbiota [7]. Black soldier fly larvae contain antimicrobial actives, such as chitin, which inhibits bacterial development and improves the function of bacteria in the gut [8]. A previous study showed that the supplementation of black soldier fly larvae could improve CD8^+^ T lymphocyte proliferation, which was beneficial for young chicks to resist avian infectious bronchitis virus infection [9]. The supply of live black soldier fly larvae has a positive effect on post-weaning piglet behavior while maintaining performance [10]. Using a black soldier fly larval meal as a protein resource in pig diets has a substantial impact on fattening pigs’ gut microbiota and colonic metabolites [11]. Recent research has revealed that the ingestion of a black soldier fly larval meal may decrease diarrhea and enhance intestinal barrier function in weaned piglets afflicted with enterotoxin-producing *E. coli* K88 [12]. Black soldier fly pulp and a black soldier fly meal were previously used as feed additives. Recently, black soldier fly extract has been developed to enrich various bioactive ingredients, which is a promising new type of nutritional additive. However, previous studies of BFE focused on weaned pigs and fattening pigs, and ignored the importance of the early stage of piglets after birth. Because PEDV causes great harm to young piglets, nutritional methods to improve the intestinal health of young piglets are crucial for later growth and resistance to microbial infections. To the best of our knowledge, whether BFE could alleviate intestinal injury in piglets infected with PEDV remains unclear.

Therefore, the present study aimed to explore whether dietary BFE can affect the growth performance, plasma biochemical parameters, antioxidant capacity, and immune function of piglets after PEDV infection. The findings of this study provide vital information for preventing and treating PEDV and may lead to new remedies for viral infections in other animal species.

## 2. Materials and Methods

### 2.1. Animal Trial

All animal study aspects were reviewed and approved by the Institutional Animal Care and Use Committee of Wuhan Polytechnic University (NO. WPU202211004). Animal welfare guidelines were followed during the trial. In all, 18 healthy 7-day-old piglets (Duroc × Landsay × Yorkshire, body weight = 2.51 ± 0.20 kg, half of the females and half of the males) were employed in this study. The pigs (three males and three females per group) were assigned randomly to the following treatment groups: control, PEDV, and BFE + PEDV (six replicates per treatment group, one pig per replicate). The pigs were housed separately in two environmentally controlled nurseries (30 ± 2 °C) with strict control of cross-contamination between the control and PEDV rooms. Free drinking water was available throughout the study period. The BFE was obtained from Proou Biotechnology (Hubei) Co., Ltd. (Ezhou, China), with a purity of 45%, about 15% of oil, and less than 15% of water content. The piglets were fed a basal feed (liquid milk replacer) from Wuhan Anyou Feed Co., Ltd. (Wuhan, China), that met or exceeded the dietary requirements of nursing piglets. During feeding, the milk replacer powder was dispersed in warm water (45–55 °C) to produce an emulsified meal with a 20% dry matter concentration. The pigs were given liquid feed every three hours from 8:00 a.m. to 8:00 p.m. during an 11-day experiment. Piglets in the BFE + PEDV group were provided 500 mg/kg BW oral BFE (dissolved in liquid milk substitute) on days 4–10 of the trial, whereas the remaining two groups got the exact same amount of liquid milk replacer. Piglets in the BFE + PEDV group and the PEDV group received PEDV at an oral dosage of 10^6^ TCID_50_ (50% tissue culture infectious dose) on day 9 of the study. An identical amount of a sterile saline solution was administered to the control group [13]. Piglets’ weights were assessed on days 1, 9, and 11 of the study, and the average daily gain (ADG) was computed.

### 2.2. Sample Collection

On the 11th day, all piglets received 10% D-xylose (1 mL/kg BW) by mouth. Piglets were weighed an hour later, and blood was drawn from their anterior vena cava to a heparinized vacuum tube (Becton–Dickinson Vacutainer System, Franklin Lake, NJ, USA). The piglets were subsequently sedated with sodium pentobarbital (50 mg/kg BW, iv), and an intestinal sample was collected. Prior to further analysis, intestinal samples were preserved in 4% paraformaldehyde, or frozen in liquid nitrogen, and kept at −80 °C [14]. In short, the pig’s belly was opened immediately after slaughter, revealing the entire gastrointestinal tract. Dissecting the intestine from the mesentery, 1 cm and 10 cm long segments from the mid-jejunum, mid-ileum, and mid-colon, respectively, were placed on a cooled stainless-steel tray [15,16]. A 5 cm section of the intestine was delicately washed with ice-cold phosphate-buffered saline (PBS) at a pH of 7.4, then submerged in a 4% solution of freshly prepared formalin for histological assessments. The 10 cm segment was opened longitudinally, rinsed with ice-cold PBS, stored at 4 °C, wrapped in tin foil, snap-frozen in liquid nitrogen, and stored at −80 °C until analyzed. Following euthanasia, all samples were collected within 15 min.

### 2.3. Blood Biochemical Measurements in Plasma

Plasma was extracted from blood samples by spinning at 3000 rpm for 10 min at 4 °C. Utilizing a Hitachi 7060 Automatic Biochemical Analyzer (Hitachi, Tokyo, Japan), the amounts of blood biochemical parameters, including total bilirubin (TB), alanine transaminase (ALT), alkaline phosphatase (ALP), total cholesterol (TC), triacylglycerol (TG), glucose (GLU), calcium (CA), creatinine (CREA), high-density lipoprotein (HDL), blood urea nitrogen (BUN), γ-glutamyltransferase (GGT), creatine kinase (CK), and lactate dehydrogenase (LDH), were determined with Wako kits (Wako Pure Chemical Industries, Ltd., Osaka, Japan) [15,17].

### 2.4. Determination of D-Xylose and Diamine Oxidase (DAO) Activity in Plasma

A colorimetric approach was used to determine the D-xylose concentration and DAO activity in plasma (Nanjing Jiancheng Bioengineering Institute, Nanjing, China. D-xylose: Catalog number A035-1-1; DAO: Catalog number: A088-1-1). The analyses were completed in line with the instructions provided by the manufacturer.

### 2.5. Antioxidant Capacity and Oxidation-Relevant Products in Plasma and Intestinal Tissue

The antioxidant enzymes and associated products were analyzed using samples of tissue from the plasma, jejunum, ileum, and colon. The total superoxide dismutase (T-SOD), catalase (CAT), malondialdehyde (MDA), and hydrogen peroxide (H_2_O_2_) levels were measured using a commercially available kit (Nanjing Jiancheng Institute of Bioengineering, Nanjing, China. T-SOD: Catalog number A001-1-2; CAT: Catalog number A007-1-1; MDA: Catalog number A003-1-2; H_2_O_2_: Catalog number A064-1-1) [18]. The tests were run three times each.

### 2.6. Intestinal Histomorphology

Intestinal histomorphology was assessed following the methodology outlined by Yi et al. [19]. In short, a fixed intestinal section was implanted in paraffin. A series of 5 μm slices were stained with hematoxylin and eosin. A light microscope (Leica Microsystems, Wetzlar, Germany) and the Leica Application Suite image processing tools (Leica Microsystems, Wetzlar, Germany) were used to analyze intestinal morphology. In each slice, ten randomly selected villi and crypts were examined at 16× magnification to determine villus height and crypt depth. The villus height was taken horizontally from the highest point of the villi to the crypt mouth, and crypt depth was taken horizontally from the pit’s base to its aperture. The results of these measurements were used to compute the villus height/crypt depth ratio and the villus surface area.

### 2.7. Real-Time PCR

The primer sequences of antiviral-related genes (IFN-β, Interferon-β; MX1, murine myxovirus resistance 1; ISG15, interferon stimulated gene 15; OASL, 2′,5′-oligoadenylate synthetase-like protein) used in this study are shown in Table 1. RNAiso Plus was used to isolate total RNA. The PrimeScript^®^RT kit with gDNA Eraser (Takara, Dalian, China) was then used to perform the process of reverse transcription. The qPCR was carried out using an Applied Biosystems 7500 Fast Real-Time PCR System, with a SYBR^®^ Premix Ex Taq™ (Takara, Dalian, China). The protocol of real time: 95 °C, 30 s; 95 °C, 5 s, 60 °C, 34 s, a total of 40 cycles; 95 °C, 15 s; 60 °C, 1 min; 95 °C, 15 s. The ribosomal protein L4 (RPL4) gene was employed as a reference in this research, and the relative expression of the gene was calculated using the 2^−ΔΔCt^ technique reported by Hou et al. [20]. Each indicator was repeated three times.

### 2.8. Statistical Analyses

The data were analyzed with the one-way ANOVA procedure in SPSS 26.0 software (SPSS Inc., Chicago, IL, USA). The Duncan method was used for multiple comparisons. The results are expressed as the mean ± SEM. The *p*-value was used as the criterion for judging the significance of the difference. *p* < 0.05 indicates significant differences, and *p* < 0.01 indicates extremely significant differences. In addition, the results of qRT-PCR were plotted using GraphPad Prism 8.0.

## 3. Results

### 3.1. Average Daily Weight Gain of Piglets

There were no differences in ADG between the groups of piglets on test days 1–8 (pre-challenge) (*p* > 0.05); Table 2. On test days 9–11 (post-challenge), PEDV infection decreased ADG (*p* < 0.05).

### 3.2. Plasma Biochemical Parameters

In comparison to uninfected piglets, PEDV-infected piglets showed greater plasma TG and BUN levels (*p* < 0.001) but decreased plasma ALP (*p* < 0.05), TC (*p* < 0.001), CA (*p* < 0.05), and CK (*p* < 0.001) (Table 3). Supplementation with BFE lowered TB but raised plasma GLU (*p* < 0.05) compared to the PEDV group.

### 3.3. Blood DAO Activity and D-Xylose Concentrations

Piglets infected with PEDV showed lower plasma D-xylose concentrations and increased plasma DAO activity compared to uninfected piglets (*p* < 0.05) (Table 4).

### 3.4. Levels of Antioxidant Enzymes and Oxidation-Related Products in Plasma and Intestinal Tissues

Compared with the control group, the viability of T-SOD in plasma, the content of MDA in the jejunum and the content of H_2_O_2_ in the colon were increased after PEDV infection, and the viability of CAT in plasma, jejunum, and colon was reduced (*p* < 0.05). Compared with the PEDV group, BFE supplementation significantly increased the activity of T-SOD in the plasma, ileum and colon (*p* < 0.05), CAT activity in the plasma and colon (*p* < 0.05), and the content of MDA in the ileum (*p* < 0.001), and significantly decreased the activity of T-SOD in the jejunum (*p* < 0.05), MDA content in the plasma, jejunum and colon (*p* < 0.05), and H_2_O_2_ content in the colon (*p* < 0.05) (Table 5).

### 3.5. Intestinal Morphology

**Figure 1:**
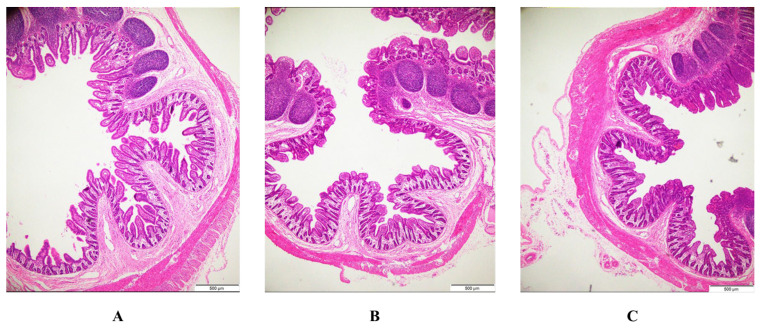
Effects of BFE supplementation on histopathological structure of the ileum. (**A**) Control. (**B**) PEDV. (**C**) BFE + PEDV (hematoxylin and eosin staining, ×16).

After PEDV infection, the ileal villi of piglets are severely atrophied or shed, and the supplementation of BFE can significantly reduce these lesions (Figure 1). Infection with PEDV reduced the villus height, villus height/crypt depth ratios, and villous surface area in the jejunum and ileum (*p* < 0.001) while increasing crypt depth in the ileum (*p* < 0.05) (Table 6). In comparison to PEDV-infected pigs, BFE supplementation raised the villus height/crypt depth ratios of the jejunum, as well as the villus height and villous surface area of the ileum, while decreasing the jejunum’s crypt depth (*p* < 0.001).

### 3.6. IFN-β, MX1, ISG15 and OASL, and Villin mRNA Levels in the Jejunum, Ileum, and Colon Tissue Samples

**Figure 2:**
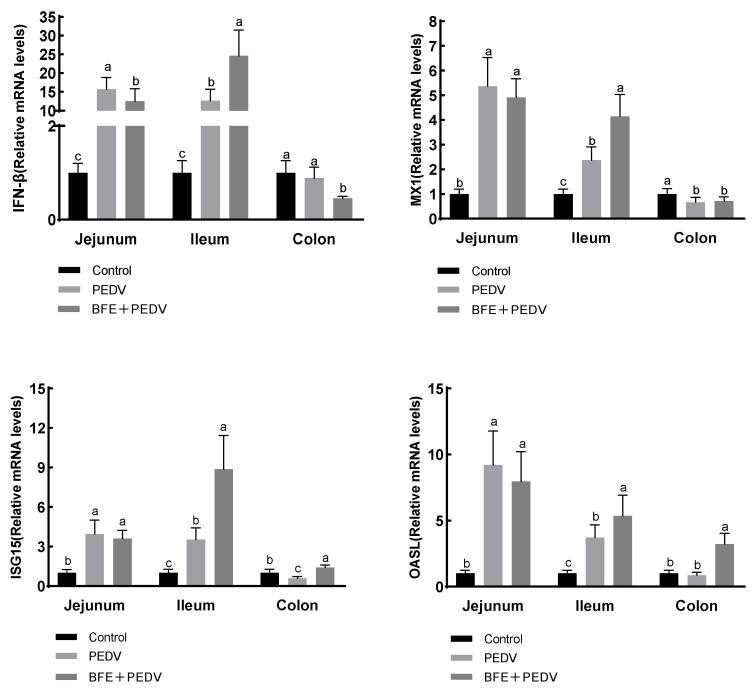
Effect of BFE supplementation on the relative mRNA expression levels of IFN-β, MX1, ISG15, and OASL in the jejunum and ileum of piglets after PEDV infection. Values are the mean and pooled SEM, n = 6. PEDV, porcine epidemic diarrhea virus; BFE, black soldier fly extract. a,b,c within a row, means with different superscripts differ, *p* < 0.05. IFN-β, interferon-β; MX1, murine myxovirus resistance 1; ISG15, interferon stimulated gene 15; OASL, 2′,5′-oligoadenylate synthetase-like protein.

PEDV-infected pigs had higher mRNA levels of IFN-β, MX1, ISG15, and OASL in the jejunum and ileum (*p* < 0.001), but lower levels of MX1 and ISG15 in the colon (*p* < 0.001); Figure 2. Compared with the PEDV group, supplementation with BFE reduced IFN-β mRNA levels in the jejunum (*p* < 0.001), raised IFN-β, MX1, ISG15, and OASL mRNA levels in the ileum (*p* < 0.001), increased ISG15 and OASL mRNA levels in the colon, and decreased IFN-β mRNA levels in the colon (*p* < 0.001).

## 4. Discussion

Porcine epidemic diarrhea virus is the leading cause of deadly watery diarrhea in pigs. The advent of highly pathogenic strains renders standard vaccinations useless in protecting piglets against PEDV infection [21]. A PEDV infection damages the intestinal health of piglets and causes enormous economic loss. Although various compounds have been studied as functional feed additives to improve the intestinal environment of animals, the prevention and control of PED remains very challenging [22]. Black soldier fly larvae provide a very nutritious diet because of their substantial amount of protein (40–44%) and a well-rounded amino acid composition comparable to, or even exceeding, that of soybean meals [23], which is considered promising feed supplementation. Recent research revealed that an alternate consumption of black soldier fly larvae reduced diarrhea and enhanced intestinal barrier function when weaned pigs were challenged with enterotoxic *E. coli* K88 [12]. Furthermore, treatment with black soldier fly larvae enhanced antioxidant capacity in broiler chickens [24]. However, BFE’s effects on PEDV-induced diarrhea and intestinal dysfunction remain unknown.

In the current investigation, a PEDV infection significantly lowered piglet ADG. This reduction appears to be closely related to PEDV-induced intestinal dysfunction, as seen by Curry et al. and Wu et al. [25,26]. The PEDV infection has been observed to produce acute atrophic enteritis and viremia [27]. According to this view, PEDV infection increases biochemical markers such as TG and BUN in pig plasma and decreases ALP, TC, CA, and CK in plasma. Similar to Zhang et al. [14], the results of the study suggest that PEDV infection makes piglets more susceptible to systemic inflammation. The BFE supplementation did not alleviate the decrease in ADG and blood indexes due to PEDV infection. In addition, the infection dose of the present study, 10^6^ TCID_50_, was higher than that in the previous study which was 10^4.5^ TCID_50_ [25]. Different doses of infection cause different degrees of morbidity and may therefore influence the effect of feed additives.

In healthy pigs, the small intestinal mucosa facilitates the absorption of D-xylose into the bloodstream. Damage to the gut impairs intestinal absorption function, resulting in a drop in D-xylose levels in the blood. Consequently, the blood’s D-xylose concentration can indicate the degree of intestinal injury and the body’s capacity to absorb it [28]. Diamine oxidase (DAO) is an enzyme that is prevalent in intestinal epithelial cells but is less active in the blood. When activated, intestinal mucosal epithelial cells release DAO into the bloodstream. Therefore, the amount of DAO activity in the blood serves as a gauge for the gut’s structural and morphological integrity in animals [29]. In this research, a PEDV infection drastically reduced the level of D-xylose while increasing the activity of DAO, showing that a PEDV infection damaged the intestinal barrier of pigs and had a negative impact on the small intestine’s absorption function. The findings match those of earlier research [30]. Supplementation with BFE had no impact on PEDV infection.

Reactive oxygen species free radicals have the capacity to damage cell macromolecules by causing oxidative stress, which results in negative biochemical responses such as protein degradation, peroxidation of lipids, mutations in DNA, and inactivation of enzymes. To protect themselves from the detrimental effects of oxygen-free radicals, cells possess a complex protective mechanism intended to neutralize the numerous free radicals created during metabolism [31]. Superoxide and dismutase (SOD) and catalase (CAT) stand out as pivotal antioxidant enzymes crucial for resisting oxidative stress. On the other hand, hydrogen peroxide (H_2_O_2_) and malondialdehyde (MDA) serve as significant indicators reflecting the presence of oxidative stress [32]. In this trial, PEDV infection reduced plasma and jejunal CAT activity while raising MDA in the jejunum and H_2_O_2_ in the colon. These results indicated that PEDV successfully induced oxidative damage to body fluids and intestinal mucosa in piglets. Notably, BFE supplementation alleviated this range of oxidative stress through increased plasma and intestinal SOD activity while decreasing MDA and colon H_2_O_2_ levels in the plasma, jejunum, and colon. In previous studies, phytophagous insects increased the expression of antioxidant enzymes such as SOD, CAT, and GPX [33]. High hydroxyl radical scavenging activity, ABTS free radical elimination capacity, superoxide free radical scavenging capacity, DPPH free radical elimination ability, and reducing power are all present in the low-molecular-weight black soldier fly larval protein hydrolysis product [34]. Kim et al. found that diets with black soldier fly larvae oil in the diet increased the antioxidant capacity of broiler serum [24]. In addition, the most recent research found that supplementing the diet with black soldier fly larvae boosted the activity of GSH in the serum of weaned pigs [35]. In short, the present study suggested that BFE could ameliorate the oxidative stress in piglets caused by a PEDV infection.

Piglets’ growth potential depends on their intestinal health. Villus height, crypt depth, and villous surface area are directly connected to absorption function and are regarded as good indexes of the small intestine structural health in animals [36]. In this research, a PEDV infection reduced villus height, villus height/crypt depth ratio, and villous surface area, and enhanced crypt depth in the jejunum and ileum, suggesting that PEDV caused intestinal structural damage and increased mucosal permeability. This is similar to the findings of our earlier investigation [13,30]. Interestingly, BFE supplementation increased villus height and the villus height/crypt depth ratio in the ileum while decreasing crypt depth in the jejunum. Fortunately, there were no negative effects on the jejunum and ileum. Similarly, after being challenged with enterotoxigenic *Escherichia coli* K88, the morphology integrity of ileum villi of the weaned pig supplemented with *Hermetia illucens* Larvae was improved, compared to the control group [12]. Furthermore, several investigations found that feeding with black soldier flies had no significant influence on the intestinal architecture of pigs [37]. This might be related to the dose and extraction of functional elements like lauric acid and chitin found in BFE. According to Liu et al., endogenous chitinase may have contributed to disparities in growth performance and intestinal health in pigs fed varied quantities of a black soldier fly larval diet [38].

IFN-β, MX1, ISG15, and OASL have antiviral properties. Notably, the MX1 gene has shown resistance against different RNA viruses, including influenza A viruses, in both pigs and wild boars [39]. The ISG15 protein experiences significant upregulation in response to robust stimulation from viral infections within the body [40]. In this experiment, IFN-β, MX1, ISG15, and OASL mRNA were all expressed higher relative to one another in the jejunum and ileum due to PEDV infection. This is consistent with the experimental findings of Wang C et al. and Wang L et al. [13,40]. In Wang et al.’s study, IFN-β and MX1 were also upregulated within 12 to 24 h of PEDV infection [41]. Moreover, orally administered with BFE stimulated the mRNA expression level of antiviral-related genes in the ileum. The increase in antiviral proteins is crucial for the early stages of a viral infection, indicating that the body’s immune system is activated to counter the virus. A previous study reported that the ileum is reported as the target tissue due to the high mRNA levels of PEDV genes [25]. As a result, enhanced mRNA transcription of antiviral-related genes in the ileum following BFE supplementation suggested a positive effect on immune function in PEDV-infected pigs. It is worth noting that BFE supplementation resulted in a decrease in the expression of IFN-β mRNA in the jejunum and an increase in ISG15 mRNA in the colon. This finding presents a novel remedy for individuals suffering from viral-induced intestinal impairment.

## 5. Conclusions

Finally, we present substantial proof for the impacts of BFE on piglet growth performance, plasma biochemical parameters and blood cell counts, antioxidant capacity, intestinal morphology, and antiviral genes. The addition of 500 mg/kg BW BFE to the feed alleviated PEDV-induced oxidative stress in newborn piglets and activated the immune system.

## Figures and Tables

**Table 1:** Primer sequences of antiviral-related genes used for quantitative PCR analysis.

Gene Name	Primer		Amplicon Size (bp)
RPL4	Forward	5′-GAGAAACCGTCGCCGAAT-3′	146
	Reverse	5′-GCCCACCAGGAGCAAGTT-3′	
IFN-β	Forward	5′-AGCAGATCTTCGGCATTCTC-3′	101
	Reverse	5′-GTCATCCATCTGCCCATCAA-3′	
MX1	Forward	5′-AGTGCGGCTGTTTACCAAG-3′	150
	Reverse	5′-TTCACAAACCCTGGCAACTC-3′	
ISG15	Forward	5′-AGCATGGTCCTGTTGATGGTG-3′	164
	Reverse	5′-CAGAAATGGTCAGCTTGCACG-3′	
OASL	Forward	5′-GGCACCCCTGTTTTCCTCT-3′	139
	Reverse	5′-AGCACCGCTTTTGGATGG-3′	

**Table 2:** Effects of BFE supplementation on average daily gain in PEDV-infected piglets.

Item	Control	PEDV	BFE + PEDV	*p*-Value
Days 1–8 (pre-challenge)				
ADG (kg)	0.124 ± 0.029	0.123 ± 0.039	0.129 ± 0.019	0.934
Days 9–11 (post-challenge)				
ADG (kg)	0.109 ± 0.025 ^a^	0.050 ± 0.058 ^b^	0.068 ± 0.060 ^ab^	0.034

Values are the mean and pooled SEM, n = 6. PEDV, porcine epidemic diarrhea virus; BFE, black soldier fly extract. ^a,b^ Within a row, means with different superscripts differ, *p* < 0.05. ADG, average daily gain.

**Table 3:** Effects of BFE supplementation on plasma biochemical parameters and blood cell counts of piglets after PEDV infection.

Item	Control	PEDV	BFE + PEDV	*p*-Value
TB (mg/dL)	0.239 ± 0.063 ^a^	0.194 ± 0.072 ^a^	0.107 ± 0.040 ^b^	0.002
ALT (U/L)	38.300 ± 7.364	42.700 ± 8.394	37.500 ± 6.091	0.315
ALP (U/L)	720.000 ± 141.966 ^a^	561.500 ± 133.213 ^b^	584.500 ± 98.806 ^b^	0.029
TC (mg/dL)	134.322 ± 23.446 ^a^	99.646 ± 17.311 ^b^	94.947 ± 10.892 ^b^	<0.001
TG (mg/dL)	31.880 ± 6.526 ^b^	45.407 ± 12.005 ^a^	46.341 ± 5.467 ^a^	0.001
GLU (mg/dL)	96.110 ± 8.478 ^b^	95.400 ± 7.415 ^b^	107.017 ± 11.594 ^a^	0.040
CA (mg/dL)	12.717 ± 0.886 ^a^	11.450 ± 0.991 ^b^	11.512 ± 0.843 ^b^	0.010
CREA (mg/dL)	0.678 ± 0.076	0.687 ± 0.050	0.753 ± 0.096	0.127
HDL (mg/dL)	19.062 ± 4.446	17.145 ± 2.689	18.082 ± 2.748	0.790
BUN (mg/dL)	2.200 ± 0.607 ^b^	6.700 ± 1.372 ^a^	5.800 ± 1.694 ^a^	<0.001
GGT (U/L)	29.000 ± 4.397	24.180 ± 6.983	29.361 ± 6.612	0.146
CK (mg/dL)	281.500 ± 65.129 ^a^	178.480 ± 33.810 ^b^	174.000 ± 38.601 ^b^	<0.001
LDH (mg/dL)	752.764 ± 112.468	686.747 ± 93.216	641.017 ± 63.573	0.086

Values are the mean and pooled SEM, n = 6. PEDV, porcine epidemic diarrhea virus; BFE, black soldier fly extract. ^a,b^ Within a row, means with different superscripts differ, *p* < 0.05. TB, total bilirubin; ALT, alanine transaminase; ALP, alkaline phosphatase; TC, total cholesterol; TG, triacylglycerol; GLU, glucose; CA, calcium; CREA, creatinine; HDL, high density lipoprotein; BUN, blood urea nitrogen; GGT, γ-glutamyltransferase; CK, creatine kinase; LDH, lactate dehydrogenase.

**Table 4:** Effects of BFE supplementation on plasma DAO activity and D-xylose concentrations of piglets after PEDV infection.

Item	Control	PEDV	BFE + PEDV	*p*-Value
D-xylose (mmol/L)	1.735 ± 0.393 ^a^	0.999 ± 0.183 ^b^	0.941 ± 0.461 ^b^	<0.001
DAO (U/L)	12.350 ± 8.000 ^b^	30.300 ± 9.450 ^a^	28.090 ± 15.190 ^a^	0.002

Values are the mean and pooled SEM, n = 6. PEDV, porcine epidemic diarrhea virus; BFE, black soldier fly extract; ^a,b^ Within a row, means with different superscripts differ, *p* < 0.05. DAO, Diamine oxidase.

**Table 5:** Effects of BFE supplementation on the redox status of the piglets after PEDV infection.

Item	Control	PEDV	BFE + PEDV	*p*-Value
T-SOD				
Plasma (U/mL)	83.49 ± 1.77 ^a^	77.34 ± 3.05 ^b^	80.75 ± 4.38 ^a^	0.001
Jejunum (U/mg prot)	312.77 ± 60.84 ^ab^	344.43 ± 48.61 ^a^	272.53 ± 25.30 ^b^	0.035
Ileum (U/mg prot)	216.73 ± 27.68 ^b^	209.73 ± 17.52 ^b^	262.32 ± 28.00 ^a^	0.001
Colon (U/mg prot)	27.94 ± 2.73 ^ab^	24.93 ± 3.59 ^b^	30.07 ± 5.51 ^a^	0.042
CAT				
Plasma (U/mL)	6.31 ± 1.45 ^a^	5.13 ± 0.85 ^b^	7.22 ± 0.84 ^a^	0.005
Jejunum (U/mg prot)	2.18 ± 0.25 ^a^	1.70 ± 0.36 ^b^	1.77 ± 0.25 ^b^	0.004
Ileum (U/mg prot)	0.97 ± 0.29	1.03 ± 0.30	1.25 ± 0.11	0.128
Colon (U/mg prot)	0.65 ± 0.16 ^a^	0.38 ± 0.10 ^b^	0.61 ± 0.19 ^a^	0.001
MDA				
Plasma (nmol/mL)	2.38 ± 0.37 ^a^	2.33 ± 0.32 ^a^	1.78 ± 0.27 ^b^	0.004
Jejunum (nmol/mg prot)	0.30 ± 0.08 ^b^	0.38 ± 0.05 ^a^	0.30 ± 0.05 ^b^	0.018
Ileum (nmol/mg prot)	0.95 ± 0.21 ^b^	1.03 ± 0.19 ^b^	1.96 ± 0.41 ^a^	<0.001
Colon (nmol/mg prot)	1.07 ± 0.25 ^a^	0.95 ± 0.26 ^a^	0.65 ± 0.16 ^b^	0.008
H_2_O_2_				
Plasma (mmol/L)	37.38 ± 7.03	41.55 ± 11.01	31.38 ± 7.14	0.104
Jejunum (mmol/mg prot)	3.66 ± 0.56	3.22 ± 0.56	3.50 ± 0.49	0.216
Ileum (mmol/mg prot)	7.77 ± 1.40 ^b^	9.13 ± 1.51 ^ab^	9.82 ± 2.06 ^a^	0.049
Colon (mmol/mg prot)	5.51 ± 1.17 ^b^	7.34 ± 2.12 ^a^	4.57 ± 1.43 ^b^	0.008

Values are the mean and pooled SEM, n = 6. PEDV, porcine epidemic diarrhea virus; BFE, black soldier fly extract. ^a,b^ Within a row, means with different superscripts differ, *p* < 0.05. T-SOD, total superoxide and dismutase; CAT, catalase; MDA, malondialdehyde; H_2_O_2_, hydrogen peroxide.

**Table 6:** Effects of BFE supplementation on the intestinal mucosal morphology of piglets after PEDV infection.

Item	Control	PEDV	BFE + PEDV	*p*-Value
Villus height (μm)				
Jejunum	291.05 ± 46.88 ^a^	170.79 ± 11.51 ^b^	191.05 ± 20.30 ^b^	<0.001
Ileum	384.06 ± 47.37 ^a^	154.55 ± 30.12 ^c^	197.17 ± 17.47 ^b^	<0.001
Crypt depth (μm)				
Jejunum	102.25 ± 15.88 ^c^	150.72 ± 15.47 ^a^	119.14 ± 12.33 ^b^	<0.001
Ileum	126.85 ± 16.45 ^b^	164.00 ± 23.96 ^a^	152.78 ± 13.64 ^a^	0.001
Villus height/crypt depth				
Jejunum	3.01 ± 0.61 ^a^	1.20 ± 0.17 ^c^	1.81 ± 0.13 ^b^	<0.001
Ileum	3.16 ± 0.46 ^a^	1.01 ± 0.20 ^b^	1.27 ± 0.11 ^b^	<0.001
Villous surface area (μm^2^)				
Jejunum	19616.55 ± 3759.46 ^a^	13014.98 ± 1822.92 ^b^	13324.72 ± 1403.05 ^b^	<0.001
Ileum	41321.35 ± 6524.20 ^a^	11069.21 ± 2375.02 ^c^	15647.26 ± 1505.07 ^b^	<0.001

Values are the mean and pooled SEM, n = 6. PEDV, porcine epidemic diarrhea virus; BFE, black soldier fly extract. ^a,b,c^ Within a row, means with different superscripts differ, *p* < 0.05.

## Data Availability

The raw data supporting the conclusions of this article will be made available by the authors, without undue reservation.
